# Classification of Retinal Diseases in Optical Coherence Tomography Images Using Artificial Intelligence and Firefly Algorithm

**DOI:** 10.3390/diagnostics13030433

**Published:** 2023-01-25

**Authors:** Mehmet Batuhan Özdaş, Fatih Uysal, Fırat Hardalaç

**Affiliations:** 1 Department of Electrical and Electronics Engineering, Faculty of Engineering, Gazi University, Ankara TR 06570, Turkey; 2 Department of Electrical and Electronics Engineering, Faculty of Engineering and Architecture, Kafkas University, Kars TR 36100, Turkey

**Keywords:** biomedical image processing, deep learning, firefly algorithm, hierarchy classification, machine learning, optical coherence tomography

## Abstract

In recent years, the number of studies for the automatic diagnosis of biomedical diseases has increased. Many of these studies have used Deep Learning, which gives extremely good results but requires a vast amount of data and computing load. If the processor is of insufficient quality, this takes time and places an excessive load on the processor. On the other hand, Machine Learning is faster than Deep Learning and does not have a much-needed computing load, but it does not provide as high an accuracy value as Deep Learning. Therefore, our goal is to develop a hybrid system that provides a high accuracy value, while requiring a smaller computing load and less time to diagnose biomedical diseases such as the retinal diseases we chose for this study. For this purpose, first, retinal layer extraction was conducted through image preprocessing. Then, traditional feature extractors were combined with pre-trained Deep Learning feature extractors. To select the best features, we used the Firefly algorithm. In the end, multiple binary classifications were conducted instead of multiclass classification with Machine Learning classifiers. Two public datasets were used in this study. The first dataset had a mean accuracy of 0.957, and the second dataset had a mean accuracy of 0.954.

## 1. Introduction

Optical Coherence Tomography (OCT) is a non-invasive biomedical imaging technique that is used to perform diagnostic imaging of the retina [1]. When compared to traditional, regular retinal fundus examinations, it has a significant advantage in detecting a wide range of retinal diseases [2] because it can capture high-resolution biomedical tissues of the retina. As this imaging technique is used for the early detection of retinal diseases, OCT images are used in this study. One of the most important areas of the retina in the OCT image is the macular area. The macular area is responsible for fine vision, color vision, and other visual functions [3]. This area is in the center of the retina, and it acts as a sensor that takes the focused light from the lens and converts it into a neural signal [4]. Retinal diseases affect the macular area, so the signal that is sent to the brain by the retina is affected as well. Therefore, this situation causes blindness, blurred vision, etc. [4]. Retinal diseases affect the macular area by changing the shapes of the retinal layers. Therefore, knowing the changes in the shape of the retinal layers is important for the detection of the disease. 

**Figure 1:**
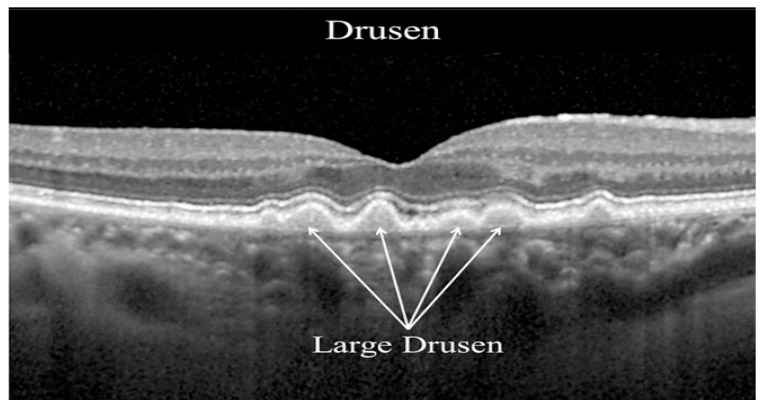
Drusen in OCT image [13].

**Figure 2:**
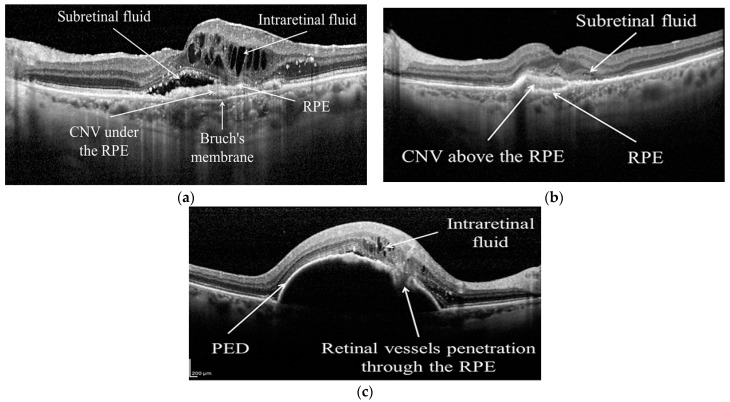
Three types of CNV disease in OCT image [13]: (**a**) Type 1 CNV; (**b**) Type 2 CNV; (**c**) Type 3 CNV.

One of the most common retinal diseases is age-related macular degeneration (AMD), which is the leading cause of blindness in people over the age of 50 [5]. According to the review conducted by Flores et al. [6], AMD was a primary reason for moderate or severe vision in 2015, and it is expected that the number of AMD diseases will be 288 million by 2040. In the early stages of the disease, dark adaptation is impaired; on the other hand, the impairment of light adaptation is minimal. However, as the disease grows, the retinal tissue degenerates and the damage becomes permanent [7]. As a result, blindness can occur. AMD has two main symptoms, which are drusen (DRUSEN) and choroidal neovascularization (CNV) [8]. These can also be called dry AMD and wet AMD. DRUSEN is a deposit that looks similar to a little bump in the Retinal Pigment Epithelium (RPE) layer. It causes the smooth shape of the RPE layer to become bumpy [9]. DRUSEN is also called early AMD, so it impairs dark adaptations, as it was mentioned before. CNV is the growth of blood vessels under or in the retina [10]. There are three types of CNV. The first type expands through the Bruch’s membrane (BM) under the RPE layer; the second type spreads into the subretinal space above the RPE layer [11]. The last type penetrates through the RPE layer [9]. All types can have subretinal or intraretinal fluids [12]. CNV is also called late AMD; it damages the retina more than DRUSEN disease. DRUSEN and three types of CNV can be seen in Figure 1 and Figure 2 [13].

**Figure 3:**
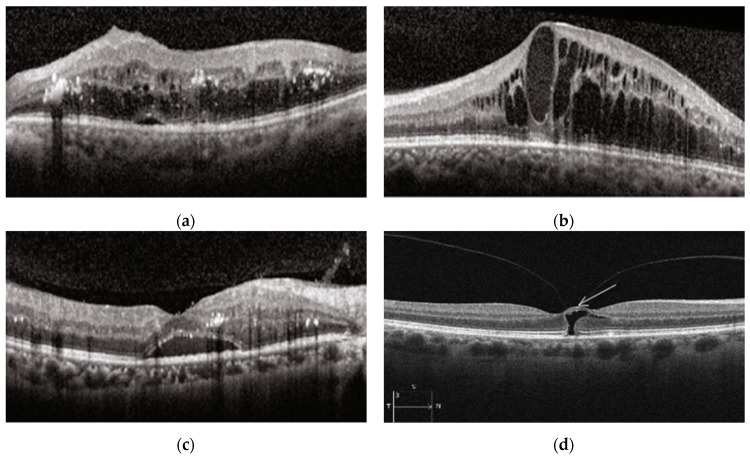
DME disease in OCT images [19,20]: (**a**) Retina thickening; (**b**) Intraretinal cysts; (**c**) Subretinal fluid above the RPE; (**d**) Vitreomacular traction.

The other most common retinal disease is diabetic retinopathy (DR), which also causes blindness. At present, this disease affects 171 million people [14]. It is expected to reach 300 million cases by 2025 and 366 million cases by 2030 [14,15]. The cause of DR is long-standing hyperglycemia, wherein retinal lesions develop [16]. One of the symptoms of DR is diabetic macular edema (DME) [15]. It is the main reason for visual deterioration in patients with diabetic retinopathy [17]. The changes in DME diseases are a thickening of the central subfield, intraretinal cysts and subretinal fluid, as CNV has, and vitreomacular traction [18]. DME and CNV diseases have similar features such as subretinal fluid and intraretinal fluid in the Outer Plexiform Layer (OPL). The difference between them is that CNV has changes in the RPE layer, while DME does not. The different types of DME disease in OCT images can be seen in Figure 3 [19,20]. As can be seen in Figure 3, all DME disease types have a smooth RPE layer.

In recent years, artificial intelligence has been very useful for diagnosing diseases in biomedical areas. Artificial intelligence includes concepts such as Machine Learning (ML), Deep Learning (DL), etc. DL gives high accuracy values for classifying problems. However, to obtain high accuracy values, DL requires a large amount of data and time for training. Thus, users who do not have high-quality equipment might have a problem with DL. Users in these conditions choose to use platforms such as Google Colab or Kaggle notebooks, which allow GPU connections through the internet. This brings another problem: limited RAM, and therefore limited data. On the other hand, ML does not require as much data and time as DL does. However, ML does not acquire high accuracy values. DL gives higher accuracy values than ML because DL has good feature extractors. The traditional feature extractors used in ML do not provide good features as DL does. 

Thus, given the problems, this study has two motivations:

- The first is to present a system that gives high accuracy, such as DL methods, with a smaller amount of data and training time, such as ML methods, to those who do not have enough quality equipment for automatic classification problems.
- The second is to classify these retinal diseases on OCT images with the help of artificial intelligence to prevent the effects that these retinal diseases cause, as well as to make things easier for clinical experts or for health centers that do not have enough clinical experts.

For the given motivations, the following methods that were used in this study are given, along with their contributions:

- First, image preprocessing techniques, such as the Bilateral filter, Contrast Limited Adaptive Histogram Equalization (CLAHE), thresholding, morphologic operations (opening and closing), and masking, were used to remove unnecessary features so the classifiers could only focus on the important ones. These techniques were applied to increase the effectiveness of the study. They are not in the system we present because they can be applied or changed for different real-world data problems.
- Classic feature extractors were used along with the pretrained DL feature extractors in this study to obtain a higher accuracy value with fewer data and training time. These feature extractors consist of the Histogram of Gradients (HOG), Gray Level Co-occurrence Matrix (GLCM), pretrained VGG16, and DenseNet121 feature extractors. The reason we use classic feature extractors is that they do not require as much data or training time. However, they do not give a high accuracy value, and they did not in our study. For this problem, we added pretrained DL feature extractors. As pre-trained models already have weights, they only need one iteration, removing the burden of training the entire model for iterations. With this method, we aimed to save training time while also obtaining a higher accuracy value.
- Even if we saved time and obtained higher accuracy with the previous method, there were so many features because so many feature extractors were used. Some of these features may not affect accuracy in a good way. Therefore, to remove unnecessary features and obtain the same or higher accuracy value with fewer data and therefore less training time, we used the Firefly algorithm for feature selection. We achieved our goal by choosing the best features with the help of this method.
- Another method we used to improve accuracy was to use multiple binary classifications rather than multiclass classifications because binary classifications give the best accuracy values in ML classifiers. If there are four classes, three binary classifications are conducted instead of one four-class classification to increase the accuracy. This classification technique is called hierarchy classification. Classic ML classifiers, which are Support Vector Machine, Logistic Regression, and Random Forest were used in hierarchy classification.

To summarize, our hybrid system consists of ML and trained DL feature extractors, the Firefly algorithm, and hierarchy classification. 

Two public datasets were used in this study. The first dataset has four classes, which are CNV, DME, DRUSEN, and NORMAL. However, the second dataset has three classes: AMD, DME, and NORMAL. The only difference between them is that in the second dataset, the AMD classes were not split into CNV and DRUSEN classes. Consequently, there are three classifications in the hierarchy of the first dataset. The first classification is between Normal and Abnormal (CNV, DRUSEN, and DME) classes. The second classification is between AMD (CNV and DRUSEN) and DME classes. The last classification is between AMD classes, CNV, and DRUSEN. Only two classifications have been conducted for the second dataset, which are Abnormal vs. Normal and AMD vs. DME classes. With Logistic Regression, a mean accuracy value of 0.957 was obtained for the first dataset, and a mean accuracy value of 0.954 was obtained for the second dataset.

The paper is organized as follows: Section 2 gives a literature summary about retinal disease classification on OCT images. Section 3 presents the methods that were used in this study. In Section 4, detailed results are shared. Discussions about the study and the literature are given in Section 5. The conclusion about the results is given in Section 6. 

## 2. Related Work

Studies in the literature can be divided into ML and DL. In ML studies, Liu et al. [21] used a Support Vector Machines classifier to classify four classes: normal macula, macular edema, macular hole, and AMD. They used 326 OCT scans that were taken from 136 subjects, and for image preprocessing, they did image alignment through thresholding, median filtering, morphological operations, curve fitting, and warping. Local Binary Patterns (LBP) were used along with multi-scale spatial pyramids (MSSP) to extract features. As they obtained so many features from the feature extraction methods, Principal Component Analysis (PCA) was used to reduce the dimensions. Instead of classifying four classes, binary classification was used as the “one vs. the rest” approach. They obtained a minimum 0.93 AUC value for all classifications and a 0.94 AUC value for the mean of them using the Area Under Curve (AUC) value.

Anantrasirichai et al. [22] also used Support Vector Machines as a classifier. The Intensity level distribution (ILB), run-length measures (RLM), GLCM, 2 levels of complex wavelet transform (CWT), LBP, and granulometry (GRA) feature extraction techniques were used. Similarly to Liu et al. [21], they used PCA to reduce the dimensions. For testing, 24 retinal OCT images were used, and an accuracy value of 0.8515 was obtained. 

Alqudah et al. [23] classified five classes, that include AMD, CNV, DRUSEN, DME, and NORMAL. They used 136,000 images for training and 1250 images for testing. They extracted features through a pre-trained DL model called AOCTNet. Then they used eight different ML algorithms: Support Vector Machines with a linear kernel, Support Vector Machines with a Radial Basis Function kernel, Artificial Neural Network, K Nearest Neighbor, Random Forest, Linear Discriminant Analysis, Quadratic Discriminant Analysis, and Naïve Bayes. They obtained the best accuracy values of 99.44 and 99.12 using K nearest neighbor and Random Forest, respectively.

The study of Srinivasan et al. [24] is another study that used Support Vector Machines as a classifier. Block matching and 3D filtering (BM3D) were used to reduce the noise in OCT images. They flattened the retinal curve and removed the unnecessary parts of the OCT images by cropping. The histogram of Gradients technique was used for feature extraction. Lastly, they classified three classes (AMD, DME, and NORMAL) with a one-to-one approach (AMD vs. DME, AMD vs. NORMAL, and DME vs. NORMAL). The images used in the study were obtained from 45 patients: 15 with AMD, 15 with DME, and 15 with NORMAL. Images from 42 patients were used for training, and images from three additional patients were used for testing. A total of 30 patients with DME and AMD were correctly diagnosed, while two NORMAL patients were not.

Wang et al. [25] used the dataset that was used in Srinivasan et al.‘s study. However, instead of classifying patients, as in Srinivasan et al.‘s study, they classified images. There were over 3000 images in the dataset, but they excluded some of them because of the irregular lighting and motion blur. Therefore, their dataset consisted of 453 AMD images, 511 DME images, and 1403 NORMAL images. Image preprocessing was conducted first in their study. Then, they extracted features using Linear Configuration Patterns along with MSSP. They used Sequential Minimal Optimization (SMO), SVM, Logistic Regression (LR), Naïve Bayes (NB), Decision Tree (DT), Random Forest (RF), and Multilayer Perceptron (MLP) classifiers for classification. The 10-fold cross-validation technique was used to evaluate the classification performance. The SMO classifier achieved the highest accuracy value of 0.9993. They did not specify the number of training and testing images.

Albarrak et al. [26] classified only two classes: AMD and NORMAL. In this study, 140 volumetric OCT images were used. They did some image processing, including retina layer extraction for encompassing the retina and warping for flattening the retina. Local histogram-based feature vectors were achieved using LBP and HOG feature extractors. Then, PCA was applied to obtain a summarized total feature vector. Lastly, the Bayesian classifier was used for classifying. The accuracy value they achieved for this study was 0.914.

Alsaih et al. [27] used two classes in their study (DME and NORMAL). They have 32 OCT volumes in their dataset: 16 for DME and 16 for NORMAL. Every volume has 128 B-scan images; thus, in total, they have 2048 images for each of the classes. For image preprocessing, BM3D filtering was used in the study to denoise the images. They then performed the retinal flattening and cropping procedures. For feature extraction, they used LBP and HOG feature extractors. The PCA and Bag of Words (BoW) techniques were used to reduce the dimensions of the feature vector. The best results they obtained were with a linear SVM, with 0.875 specificity and sensitivity values.

Santos et al. [28] developed a different feature extractor technique to classify the AMD classes. They constructed a topographic map of the macular region. In addition, they computed the geostatistical features using the semivariogram and semimadogram functions, according to the topographic map. The features that were computed were used as the input for the SVM classifier. They had 384 OCT volumes: 269 for the AMD class and 115 for the NORMAL class. They achieved an accuracy of 0.952. A literature review of ML studies is summarized in Table 1.

The literature gap in ML studies is given below:

- Some of the studies do not have high accuracy values.
- The ones that have high accuracy values either have too many images, classify just two classes, or do not specify the training or testing image number they used.
- Using so many images for training takes a lot of time, a known fact in artificial intelligence studies.
- By classifying just two classes, high accuracy values can be achieved easily.
- If the number of training and testing images is not specified, no reliable comment can be made about the results in this situation.

Until now, ML studies have been examined. The number of DL studies in the literature exceeds that of ML studies. If DL studies are examined, A P et al. [29] developed a Convolutional Neural Network (CNN) model called OctNET. Their dataset contains 83,484 images, of which 37,205 are CNV images; 11,348 are DME images; 8616 are DRUSEN images; and 26,315 are NORMAL images. In addition, for testing, they used 968 images that had 242 images for each class. They achieved an accuracy of 0.9969.

Fang et al. [30] used the same dataset as A P et al., but with minor differences. Their dataset had 84,484 OCT images with four classes: 37,455 CNV, 11,598 DME, 8866 DRUSEN, and 26,565 NORMAL. They created a CNN model that combines the features from the current layer and previous layers. They achieved a 0.8773 overall accuracy value.

Das et al. [31] proposed a new model called the Deep Multi-scale Fusion Convolutional Neural Network (DMF-CNN). They used two datasets in the study. The first dataset is the same as the one used by Fang et al. The second dataset contains 1296 AMD images, 1086 DME images, and 1578 NORMAL images. They achieved a 0.9603 overall accuracy value for the first dataset and a 0.9966 overall accuracy value for the second dataset.

Huang et al. [32] classified four classes (CNV, DME, DRUSEN, NORMAL) with their proposed model, called a “novel layer guided convolutional neural network” (LGCNN). Their model includes the ReLayNet model, which segments the retinal area so that useful information can be extracted. From this RelayNet, two segmented images were obtained. Therefore, they used two subnetworks that used one image from these two images as the input. Lastly, they integrated the information that was obtained from the two subnetworks. They used two datasets. The first dataset is the same dataset that Fang et al. and Das et al. used. The second dataset includes 1581 CNV, 4592 DME, 1563 DRUSEN, and 1168 NORMAL retinal images. They obtained accuracy values of 0.88 for the first dataset and 0.99 for the second dataset.

Das et al. [33] proposed a model that imitates ophthalmologists’ way of diagnosing by focusing on the clinically informative. The dataset they used had 269 AMD patients and 115 NORMAL patients (OCT volumes). Every volume has 100 images. The second dataset they used is the same as Huang et al.‘s second dataset. In the second dataset, they had 48 AMD, 50 DME, and 50 normal subjects. In addition, the number of images in each subject range between 19 and 61. However, they down-sampled all the volumes to just 19 images. Flattening, cropping, and resizing were used for image processing. The first dataset had an accuracy value of 0.971, and the second dataset had an accuracy value of 0.932.

Another CNN model was developed by Ai et al. [34]. It is called a fusion network (FN)-based retinal OCT classification algorithm (FN-OCT). They used a dataset that has four classes (37,205 CNV, 11,348 DME, 8616 DRUSEN, and 51,140 NORMAL images). For testing, they used two datasets. One of them, which is relevant to the dataset they used for training, includes 250 images per class, or 1000 in total. The other one, which has 60 CNV, 107 DME, 27 DRUSEN, and 83 NORMAL images, is an external dataset provided by Beijing Chao-Yang Hospital. For the first test dataset, they achieved accuracy values of 0.98, 0.984, and 0.987 for the first, second, and third fusion strategies, respectively. In addition, for the external test set, they achieved 0.85, 0.8, and 0.92 accuracy values for the first, second, and third fusion strategies, respectively.

Kim and Tran [35] extracted the retina layer by segmenting the area. In addition, they used U-Net with the Fully Connected Layer for this purpose. After segmenting the retinal area with U-Net, they proposed two different architectures. The first architecture employs the “one vs. one” method. The second architecture employs the “one vs. other” method. Their dataset is the same dataset that Ai et al. used, including the first test dataset. However, they only used 34,464 images for training. Using the first architecture, they achieved a 0.981 accuracy value. In addition, in the second architecture, a 0.987 accuracy value was reached.

Li et al. [36] simply used a transfer learning approach with a pre-trained VGG16 model. They classified four diseases with the numbers 37,456 CNV, 11,599 DME, 8867 DRUSEN, and 51,390 NORMAL images. Overall, 1000 images, 250 per class, of this dataset were used for validation. Testing dataset numbers is the same as the validation dataset. They achieved 0.986 accuracy, 0.978 sensitivity, and 0.994 specificity values.

Another study that used a transfer learning approach is Asif et al.‘s study [37]. They used the ResNet50 model as a feature extractor and achieved a 0.994 accuracy value with four classes (CNV, DME, DRUSEN, and NORMAL). Their dataset is the same as that used by A P et al., including the test dataset.

Rong et al. [38] proposed a different technique, which involves generating surrogate images to train the CNN model they created. A large number of regions were cropped from the images, and the features were extracted from these cropped areas. This way, they augmented the data differently. Before they did that, image denoising was performed. To eradicate the background features in OCT images, they created a mask with thresholding and morphological operations. Finally, after generating surrogate images, they trained the CNN model. They used two datasets. The first dataset was collected from seven patients with CNV or NORMAL classes, and every patient had 12, 13, or 14 OCT volumes. A second dataset was acquired from 45 patients (15 NORMAL, 15 AMD, and 15 DME). They created the test set by taking one volume from each patient. The training and validation sets constitute the rest of the dataset (%20 for the validation set). Their method had an AUC of 0.9783 for the first dataset and 0.9856 for the second.

The study of Tayal et al. [39] is a rare study that used image processing techniques in DL studies. They used a couple of image processing techniques to remove unnecessary backgrounds from the images. Medium filtering, CLAHE, thresholding, morphological operations, and contour extraction were used for extracting the retinal layers. They used three CNN models to classify four diseases (CNV, DME, DRUSEN, and NORMAL). CNN models have five, seven, and nine layers, respectively. Their dataset consists of 37,205 CNV classes, 11,348 DME classes, 8616 DRUSEN classes, and 26,315 NORMAL classes. They split the data into three groups: training, testing, and validation, with a ratio of 90.16:1.84:8.00. They achieved 0.965 accuracy, 0.96 sensitivity, and a 0.986 specificity of values. A literature review of DL studies can be summarized in Table 2.

The literature gap for DL studies is given below:

- Almost all DL studies had a high level of accuracy. However, they had to use a lot of data.
- In addition to using a large amount of data, they had to create CNN models with a minimum of seven layers. When the number of neurons in the layers is considered, high-quality equipment is required to handle the computational load, which is why they all use GPUs.
- Training with so much data and computational load requires a lot of time for CPU users.
- Given the literature gap in both ML and DL studies, the goal of this study is to:
- Developing a hybrid system with the same high and close accuracy as DL studies
- Achieving this high accuracy value in a short time and with fewer data, such as in ML studies.
- Conducting this study using a CPU and providing a system with a low computational load for those who do not have enough computational load or data.

## 3. Materials and Methods

### 3.1. Database

We used two public datasets in our study. The first dataset was taken from the Kaggle source [40]. Our first dataset has four classes: CNV, DME, DRUSEN, and NORMAL. Every class has 7000 images for training, 242 images for testing, and 1000 images for validation. In total, there were 28,000 images for training, 986 images for testing, and 4000 images for validation. However, we did not use all of the images in the first dataset because the inclusion of so many images can affect the computing, time, and RAM.

The second dataset was taken from Srinavasan et al.’s study [24]. This dataset only has three classes, which are AMD, DME, and NORMAL. The number of patients for these classes is 15 for every class, or 45 in total. The AMD class contains 723 images; the DME class contains 1101 images; and the NORMAL class contains 1407 images. We split the images into three categories: training, testing, and validation.

Hierarchical classification is used in our study. We divide the datasets into ABNORMAL and NORMAL classes. ABNORMAL classes are CNV, DME, and DRUSEN classes for the first dataset, and AMD and DME for the second dataset. We mentioned that CNV and DRUSEN diseases are the symptoms of AMD disease. Thus, in the first dataset, we fused CNV classes with DRUSEN classes and labeled them as AMD. After that, we classified the AMD and DME classes for the first dataset. In the second dataset, there were no CNV or DRUSEN classes; therefore, we directly classified the AMD and DME classes. For the first dataset, there is only one classification left: the CNV and DRUSEN classes. Finally, we classified these classes. Table 3 contains the information about the first dataset.

In total, 3000 images for CNV, 3000 images for DRUSEN, 4000 images for DME, and 3600 images for NORMAL classes were used for training. For testing, 968 images were used in total. Some of the images are used again for different classifications, both for training and testing. The number of images that were used for the ‘AMD vs. DME’ classification is higher than the others because it was the hardest classification to classify in our experiments. Some of the images in the second dataset are removed to balance the dataset according to the classifications. Details of the second dataset are given in Table 4.

### 3.2. Image Processing

OCT images have unnecessary features for models in the background, such as white areas or noises. To remove background from images, some image processing techniques were used in our study. To extract the retinal layers, a binary matrix should be created. Thus, if a binary matrix that has a value of “1” in the area of the retinal layers is created, the retinal layers can be easily extracted by multiplying the binary matrix with the original image. For this, thresholding can be used. However, with thresholding operations, some noises in the background can be extracted as well. 

First, a bilateral filter is used to remove noise from the background. A bilateral filter takes a weighted average of the neighborhood of the central pixel and preserves the edges. It is similar to Gaussian smoothing. A bilateral filter preserves the edges, whereas a Gaussian filter does not. As Gaussian only takes the weighted average of the neighborhood, the bilateral filter considers the difference between the values in the neighborhood [41]. It is important to preserve the edges of the retinal layers in order to diagnose diseases; therefore, a bilateral filter is chosen for this purpose. (Mathematical expression in Appendix A).

To improve the result of the thresholding operation, a Contrast Limited Adaptive Histogram Equalization (CLAHE) is used. This operation improves the contrast of images, so retinal layers’ pixels can have higher values and can be extracted more easily. CLAHE is the extension of Histogram Equalization. Histogram Equalization is a contrast enhancement technique that increases the contrast in images. To achieve this, it uses a histogram of images. Histogram equalization consists of a couple of steps. First, the cumulative distribution function (cdf) is calculated (Mathematical expression in Appendix A). Lastly, the histogram value of every pixel is calculated according to the cdf values of every pixel (Mathematical expression in Appendix A). The problem with histogram equalization is that the contrast increases a lot. Therefore, CLAHE uses adaptive histogram equalization to solve this problem, which simply splits the image into blocks and applies histogram equalization to every block. However, the problem with adaptive histogram equalization is that noises amplify and become clear. CLAHE’s final method for dealing with this issue is limiting the contrast. A value is defined to limit the histograms of an image before calculating the cdf values to reduce the noise amplification. 

After these operations, thresholding is applied to the images. Thresholding operations are used for isolating an object of interest from a background. To achieve this operation, a value is chosen to segment the image. Values that are smaller than this chosen value take a 0 value, and values that are bigger than this chosen value take a 1 value. The chosen value affects the quality of thresholding. Thus, this value is generally chosen according to the images. For this, the OTSU method was used in this study.

Even if some operations are used before thresholding to obtain retinal layers, there can still be some noise in the background. For this, morphological operations—opening and closing—are used. Morphological operations deal with the shape of the features in an image. The purpose of these operations is to eliminate defects that occurred during the thresholding operations. The operations that are used in this study are opening and closing operations. Both operations consist of other morphological operations, called erosion and dilation. The difference between them is the order of application for erosion and dilation operations. In opening operations, erosion is applied before dilation. In closing operations, erosion is applied after dilation. Dilation enlarges objects, while erosion shrinks them. These two operations use structuring elements to change the shape of an object in the image. These elements can be any size and shape. For the dilation operation, if one of the elements of the structuring element touches the object in the image, the (x, y) pixel, which is the center of the structuring element, obtains the value of “1” in the output image. For erosion, the only difference is that all of the elements of the structuring element must touch an object in the image for the (x,y) pixel to obtain a value of “1” in the output image. With the opening operation, erosion is applied first to remove defects in the image. However, with erosion, the object shrinks. To obtain the original object, dilation is used after erosion. As there are no defects in the image because of erosion, these defects cannot be removed after the dilation operation. Following the opening operations, closing operations are used in the study to eliminate the problems caused by the opening operations.

After the thresholding and morphological operations, a binary image is created that has the value of “1” for the area of retinal layers. Then, this binary image is multiplied by the original image. As a result, unnecessary features are removed from the original images.

**Figure 4:**
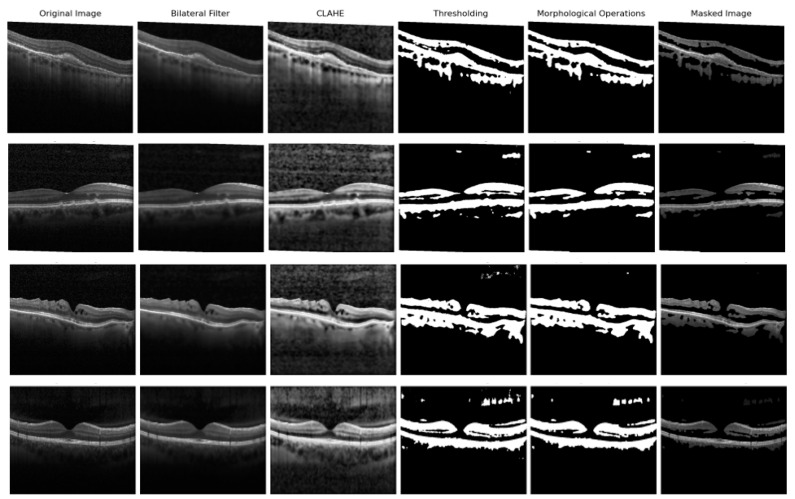
Image processing results (CNV images: First row, DRUSEN images: Second row, DME images: Third row, DRUSEN images: Fourth row).

The only thing that remains is multiplying the binary matrix by the original image. As a result, images with the retinal layers extracted are obtained. The results of these five steps are given in Figure 4. To summarize, image preprocessing consists of five steps:

1. Bilateral filter to remove background noises.
2. CLAHE operation to enhance the contrast.
3. Thresholding operation to create a binary image.
4. Morphological operations to remove noises in the binary image
5. Masking operation, multiplying the binary image with the original image.

### 3.3. Feature Extraction

Classic feature extractors and TL extractors were used for feature extraction in our study. The reason we combine them is because classic feature extractors are not enough to obtain a high accuracy value. For this problem, we could have used TL extractors only. However, we wanted to obtain the accuracy value as high as we can, so combining them and choosing the best features among them helps our cause. In total, four feature extractors were used in this study, which are given below:

- Gray Level Co-occurrence Matrix (GLCM)
- Histogram of Gradients (HOG)
- VGG16 feature extractor
- DenseNet121 feature extractor

GLCM is a second-order statistical texture analysis method. It is a matrix, and its rows and columns correspond to the gray levels in the image [42]. For example, if an image has 256 gray levels, GLCM will have 256 rows and columns. When creating the GLCM, two parameters are chosen. They are the orientation and distance parameters. Orientation is usually chosen as one of four values, which are 0°, 45°, 90°, and 135°. For example, the distance is chosen as “2” and the orientation is chosen as 45°. If there are 25 values for (2, 4), the distance between them is 2, and the orientation is 45 degrees, then the value 25 is assigned to the second row and fourth column of the GLCM. After the GLCM is created, some features are calculated from the GLCM. There are lots of GLCM features in the literature, but we only used some of them, which are energy, correlation, homogeneity, and contrast (Mathematical expression in Appendix B). 

The purpose of HOG is to characterize objects in the image by the distribution of local intensity gradients or edge directions [43]. In other words, it is to describe the image with local histograms. The HOG feature extractor consists of four methods [44]. Horizontal and vertical gradients are calculated through a convolution operation between the image and the kernel matrices. The gradients’ angle and magnitude are computed from these gradients (Mathematical expression in Appendix B). Then, the image is divided into 8 × 8 cells. Each cell will have a histogram calculation. For this purpose, nine bins are selected, usually between 0° and 180°, with a 20° difference between each bin. The magnitude values are added to this bin according to their angle values. For example, if a pixel in the cell has a magnitude of “4” and an angle of “20°,” the “4” value is added to the “20°” bin. Lastly, four cells are combined to form 2 × 2 blocks. As there are eight rows and eight columns for one cell, these blocks will have 16 rows and 16 columns. The stride value for creating blocks is one cell, so this means there can be the same cells in different blocks. For every block, L2 normalization is conducted, and as a result, the HOG feature vector is created.

Fully connected layers for pretrained VGG16 and DenseNet121 models are removed, and these models are used as feature extractors. Only one iteration is required to obtain the features because these models are already trained through the ImageNet dataset.

### 3.4. Feature Selection

We combined four feature extractors; 33,000 features are acquired with these four feature extractors. To decrease the timing in training for classification and to obtain the best features from every feature extractor, the Firefly optimization algorithm is used in this study. The Firefly algorithm was developed for continuous optimization problems [45]; as we need it for feature selection, the binary Firefly algorithm is used. The purpose of this algorithm is to obtain the highest accuracy value with the fewest features.

#### Binary Firefly Algorithm

The binary firefly algorithm is an optimization algorithm that mimics the behavior of fireflies [46]. There are some rules that describe the behavior of fireflies, which are given below [47]:

1. Regardless of gender, all fireflies will be attracted to one another.
2. Each firefly is attracted to the others by their brightness and the distance between them. Less-bright fireflies will move to the brighter fireflies. Attractiveness decreases when the distance increases between fireflies. If one firefly is the brightest, then this firefly will move randomly.
3. Firefly brightness is determined by fitness function.

The steps of our binary firefly algorithm inspired by Maza and Zouache’s [46] study and Emare et al.‘s [47] study are given below:

1. We generated a firefly population that has 20 fireflies, which means 20 subsets of features and 33,000 values (There are 33,000 features in our dataset). These 33,000 values are made up of “0” and “1” values. “0” represents an unselected feature, and “1” represents a selected feature. There is an example of this firefly population in Table 5. In Table 5, f stands for fireflies (subset features), F stands for features in the dataset. For instance, as seen in Table 5, $F_{1}$, $F_{3}$, and $F_{4}$ features are not selected for $f_{1}$ firefly, which means these features are removed from the dataset when training or testing for only $f_{1}$ firefly.
2. Every firefly (subset features) has a fitness function value that is given in Equation (1) [47].
  (1) $$fitfunc_{i}=w*acc\left(f_{i}\right)+\left(1-w\right)*\frac{\sum C_{i}==1}{\sum C_{i}},$$
  In Equation (1), w is a constant, and $acc\left(f_{i}\right)$ is the accuracy value of the i^th^ firefly (i^th^ subset features). The K-Nearest Neighbor classifier is used to obtain the accuracy values. For training, a validation set is used; then, with a testing set, the accuracy values are acquired for all fireflies. The term ‘$\sum C_{i}==1$’ is the total number of selected features of the i^th^ firefly and the ‘$\sum C_{i}$’ term is the total number of features of the i^th^ firefly, which is equal to the total number of features in the dataset (33,000).
3. Now we have a fitness function value for all of the fireflies. That means we have brightness values for all of the fireflies. Now every firefly will move to a brighter firefly. A “i” firefly, which has a lower fitness function value (brightness value), moves toward a “j” firefly, which has a higher fitness function value with Equation (2).
  (2) $$X_{i_{new}}=\beta *\left(X_{j}-X_{i}\right)+\alpha *\left(R-0.5\right),$$
  In Equation (2), X values are the position values, which have 33,000 values that consist of “0” and “1” values. R is a random number between zero and one, and α is a randomization parameter that defines the random movement of the firefly, which is determined by the users. β is an attractiveness value, and its formula is given in Equation (3).
  (3) $$\beta =\beta_{0}*e^{-\gamma *r},$$
  In Equation (3), r is the distance between two fireflies and is given in Equation (4) [46]. $\beta_{0}$ is determined by the users and defines the value when r is equal to zero. γ value is a light absorption coefficient, which is also determined by users.
  (4) $$r=\frac{\sum X_{i}-X_{j}}{2*\sum C_{i}-\left(\sum C_{i}==1+\sum C_{j}==1\right)},$$
  If “i” firefly is brighter than “j” firefly, then “i” firefly will move randomly. For this condition, new position values for the “i” firefly are calculated according to Equation (5).
  (5) $$X_{i_{new}}=X_{i}+\alpha *\left(R-0.5\right),$$
  As seen in Table 5, the position values of the fireflies must consist of “0” and “1” values to determine which features will be selected. At the end of the third step of the algorithm, new position values can be decimal values or may be outside the 0–1 range. With these new position values, we cannot tell if a feature is selected or not for i^th^ firefly. To address this problem, first the position values are drawn to the 0–1 range with Equation (6) [46], then Equation (7) is applied [46,47].
  (6) $$X_{i_{norm}}=\tanh\left(X_{i_{new}}\right),$$
  (7) $$X_{i_{final}}=\{\begin{array}{c} X_{i_{norm}}<0.5,\;0\\ X_{i_{norm}}\geq 0.5,\;1 \end{array},$$
4. After obtaining the final position values for i^th^ firefly, the new fitness function value for i^th^ firefly is calculated according to Equation (1); i^th^ firefly and all other fireflies in the population go through the same processes. As we have 20 fireflies in the population, this means i^th^ firefly’s position values and fitness function value will change 20 times.
5. The algorithm continues from step one with a new firefly, which is ${\left(i+1\right)}^{th}$. The same processes are conducted between the ${\left(i+1\right)}^{th}$ firefly and all of the other fireflies in the population.
6. When all of the fireflies’ (${\left(i+2\right)}^{th}$, ${\left(i+3\right)}^{th}$, …) position values and their fitness function values from these position values are calculated, a new iteration begins.
7. When maximum iteration is reached, a firefly that has acquired the maximum fitness function value is selected to be used in classification. The pseudo code of Binary Firefly algorithm is given in Algorithm 1.
**Algorithm 1 Binary Firefly Algorithm** **Input:** Firefly population as in Table 5 (with their fitness function value calculated by Equation (1)) **Output:** Firefly (subset features) with maximum fitness function value **while** iteration < maximum iteration  **for** i = 1 to *n* (*n* = number of fireflies)   **for** j = 1 to *n* (*n* = number of fireflies)    **if** ${\mathrm{fitfunc}}_{i}<{\mathrm{fitfunc}}_{j}$     move i^th^ firefly towards j^th^ firefly with the Equation (2)     apply Equation (6)–(7)    **else**     move i^th^ firefly randomly with the Equation (5)     apply Equation (6)–(7)    **end if**    calculate new fitness function value of new i^th^ firefly with the Equation (1)   **end for**  **end for**  rank the fireflies (subset features) according to their fitness function values **end while** Obtain the firefly (subset features) which has the maximum fitness function value

### 3.5. Classification

**Figure 5:**
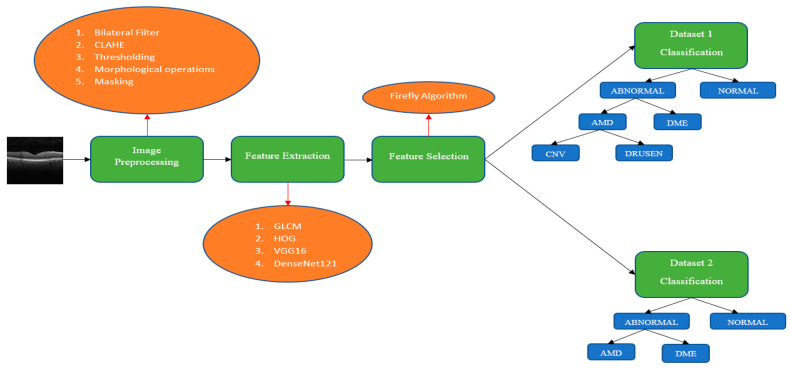
Process flow diagram.

Hierarchy classification is conducted to obtain better results. For the first dataset, three classifications are conducted. The first classification is between the abnormal and normal classes. The abnormal classes consist of the CNV, DRUSEN, and DME classes. The second classification is between the AMD and DME classes. The AMD classes consist of the CNV and DRUSEN classes. The only classification left is between the CNV and DRUSEN classes, which is the third classification for the first dataset. For the second dataset, two classifications are conducted because there are only three classes. The only difference between this dataset and the first is that it lacks CNV and DRUSEN classification. The rest is the same as the first dataset. Three classifiers are used in our study: Support Vector Machine (SVM), Logistic Regression (LR) and Random Forest (RF). The reasons behind using these methods are given below:

- Image processing techniques are used to extract retinal layers, so feature extractors can only focus on the necessary features.
- Hybrid feature extractors are used for better accuracy. Each one of them is not enough alone for better accuracy. As we were going to use the Firefly algorithm for feature selection, we decided to use them all.
- The reason we used so many feature extractors is to choose the best features among the ones they extracted. For this method, we were going to be able to take advantage of every feature extractor. As this study’s aim is to obtain the highest accuracy value with the least amount of data and training time, using the Firefly algorithm was inevitable for our study because this method can choose the best features for the best accuracy. By selecting the best features, we would reduce the amount of data for the classifiers and benefit from the best practices of all of the feature extractors.
- Although we used some techniques to improve the accuracy, for training time, we had to use ML classifiers. In addition, these classifiers do not give a high accuracy value for multiclass classification. Therefore, we decided to use the hierarchy classification technique as some of the classes include other classes, such as the ABNORMAL class, which includes the AMD and DME classes, or the AMD class, which includes the CNV and DRUSEN classes. In this way, we turned our classification system into a multiple-binary classification system, which has a higher accuracy value. This system is different from “one vs. one” or “one vs. other” classification systems because unnecessary classifications are conducted in those systems, such as DME vs. NORMAL. Instead of classifying the DME and NORMAL classes, we classify the ABNORMAL and NORMAL classes as ABNORMAL includes DME. A process flow diagram about the methods used in the study is given in Figure 5.

## 4. Results

The result metrics used in this study are given in Equations (8)–(11):

(8) $$Accuracy=\frac{TP+TN}{TP+TN+FP+FN},$$

(9) $$Precision=\frac{TP}{TP+FP},$$

(10) $$Recall=\frac{TP}{TP+FN},$$

(11) $$F1-score=2*\frac{Precision*Recall}{Precision+Recall},$$

TP means True Positive, TN means True Negative, FP means False Positive, and FN means False Negative. The results of the study are given in Table 6.

**Figure 6:**
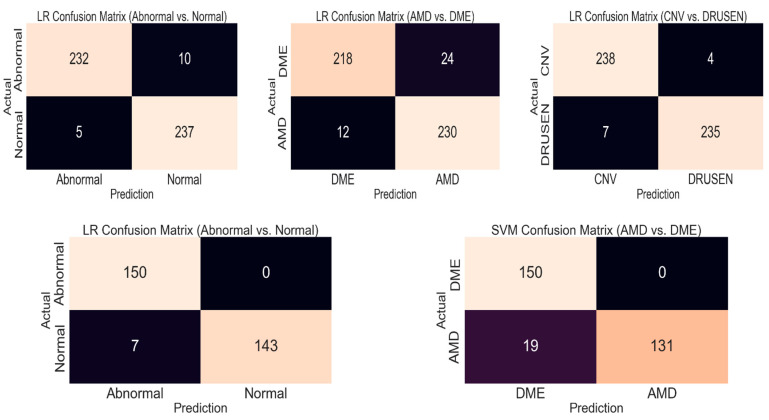
Confusion matrices for the best models (First row for the first dataset and second row for the second dataset).

Logistic regression acquired the best accuracy value for all of the classifications, except for the AMD vs. DME classification in the second dataset, for which SVM gave the best accuracy. The precision, recall, and f1-score values are calculated class by class. For example, when calculating the precision, a precision value for an abnormal class means that an abnormal class is taken as a positive class, while other classes are taken as negative classes. The confusion matrices of the models that achieved the best results are given in Figure 6.

**Figure 7:**
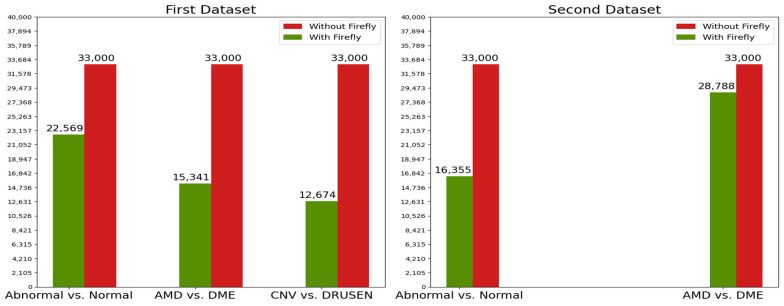
Effect of the Firefly algorithm on feature numbers for both datasets.

We mentioned that the reason we use the Firefly algorithm is to obtain the same or better results with less training time and less computing load. We also mentioned that the Firefly algorithm achieves this by reducing the features of the dataset, in other words, by removing useless features while keeping useful ones. The number of features that are used after and before the Firefly algorithm is given in Figure 7. Table 7 shows the effect of the Firefly algorithm on the model accuracy and training time. It can be seen in Figure 7 and Table 7 that the Firefly algorithm gives better or similar results with less training time and less data.

**Figure 8:**
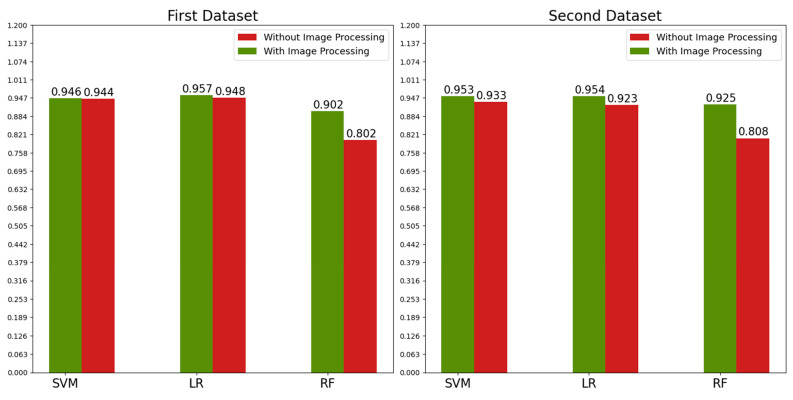
Effect of the Image processing techniques on mean accuracy value for both datasets.

Apart from the Firefly algorithm’s effects on the models’ accuracy and training time, we also examined our image preprocessing techniques’ effects on the models’ accuracy. Table 8 summarizes the effects of the image processing technique on the accuracy values for all of the classifications. According to Table 8, image processing has a positive effect in most classifications. This can be seen more clearly if we look at the average accuracy values in Figure 8.

## 5. Discussion

In this section, the effects of the different methods used in the study on the results, the reasons why these methods were chosen, and the difference between the results obtained in the study and the literature, are presented.

**Figure 9:**
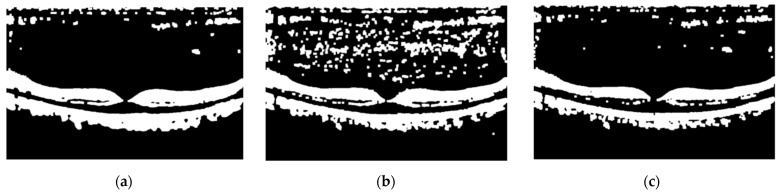
Effect of filtering techniques on masking images: (**a**) Bilateral filter; (**b**) Median filter; (**c**) Gaussian filter.

Methods in image preprocessing are basic techniques that are used for most classification problems. Normally, after the thresholding and morphological operations, contour detection is used, but we used a masking operation, which gives the same results and is faster and easier to apply. Another technique we can change from these methods is the kind of filtering that can affect later operations. First, we used two filters: the median, and Gaussian filters. These filters are effective, but they blur the edges, which are critical in this study. Therefore, we used three other filters, which are bilateral: BM3D and the non-local (NL) means filter. The BM3D and NL-means filters outperformed the bilateral filter, but they were slower. Thus, we decided to use a bilateral filter. It blurred the noise while protecting the image’s edges in a short amount of time. Image processing is used in a few studies in the literature. Liu et al. [21] used some of the same image processing techniques, such as thresholding, but only for retinal layer alignment. For retinal layer extraction, they used edge detection techniques, but with edge detection techniques, images do not become as clear. Srinivasan et al. [24] used a better filter for denoising, which is BM3D filtering, as we mentioned, and for retinal layer extraction, they cropped the images. BM3D filtering takes time, and when using sufficient images, this time increases a lot. Cropping images reduces the amount of data, so using this technique with a faster filter would be very beneficial for our study, but as they mentioned in their study, cropping images has the possibility of missing the pathology appearing in the more important regions. Albarrak et al. [26] and Alsaih et al. [27] also used these denoise and crop image processing techniques. The image processing techniques in the Tayal et al. [39] study are similar to ours. They used medium filtering for denoising, thresholding, morphological operations, and contour extraction for retinal layer extraction. Medium filtering is faster than BM3D filtering, but it is not the best filtering technique. Therefore, instead of a slow technique, such as BM3D, or a technique that does not achieve the best results, such as medium filtering, we preferred to use the bilateral filter, which is a good and fast filtering technique. Contour extraction is not necessary; a masking operation can simply be applied instead of contour extraction for retinal layer extraction. In Figure 9, the effect of the filtering techniques on the masking images is shown.

The filtering techniques shown in Figure 9 are the quickest, and a bilateral filter is better than the others for removing noise. From the given discussion of image processing techniques, the differences from the literature can be summarized as below:

- The studies that used filtering techniques used BM3D and medium filtering. BM3D filtering is very good but not very fast. The filtering technique we used, bilateral filter, is enough to keep the important areas of the retinal layers, while removing noise better than a medium filter, and it is faster than BM3D filtering.
- Lots of ML studies used cropping for retinal layer extraction, but it may cause important features to be overlooked in some images, which affects the accuracy in a bad way. Our image processing techniques do not have this problem, as we successfully extracted the retinal layers by using every part of the image.

For feature extraction, in the literature, either DL models were trained, which takes a lot of time, or basic feature extractors were used, which do not give high enough accuracy values. We used a hybrid feature extractor that combines basic feature extractors with pre-trained DL feature extractors, which is transfer learning. Normally, basic feature extractors do not give high accuracy values. However, there is one study that acquired a high accuracy value with those extractors, and that is the Wang et al. [25] study. However, they did not give specific details about the testing images they used, which can affect the accuracy. There are three studies in the literature that used transfer learning for feature extraction. Using transfer learning is good for reducing the training time, but these three studies used a lot of data for this, which eliminates this advantage. Alqudah et al. [23] used AOCTNet, which is trained with OCT images. This feature extraction technique achieves good results for OCT images, but we want to present a system that can be used for all diagnosing systems, not just retinal diseases. As a result, using AOCTNet to diagnose various problems may yield poor results. The differences between our hybrid feature extraction system and others can be summarized as follows:

- DL studies train their own models, which is time-consuming. Our hybrid feature extractors do not require as much time.
- Using only basic feature extractors does not give the high accuracy values that are used in ML studies. By combining basic feature extractors with transfer learning, our hybrid feature extractors can provide high accuracy with limited data and a short amount of time.

For DL studies, there are no feature selection algorithms, which is quite normal. If we look at the studies that used feature selection algorithms, all of them used the PCA algorithm. The PCA algorithm is good for reducing the dimensionality of the data and can reduce the training time for classifiers. However, to obtain the same or better accuracy value as the original data, not too much reduction should be conducted. Thus, instead of changing the little reduction in the data, we preferred to use the Firefly algorithm, which has the ability to give the same or similar accuracy value even though it reduces the data by half, as can be seen in the “Results” section. The reason for this is that PCA gives a general idea of the data without knowing its accuracy value, while the Firefly algorithm chooses the features by obtaining the accuracy values from these features, which is much more precise for diagnosing problems.

To improve the accuracy, we use hierarchy classification. There is no study in the literature that uses hierarchical classification. Some of the studies used “one vs. one” or “one vs. other” methods, but there are some unnecessary classifications when these methods are used, as we mentioned in the “Classification” subsection in the “Materials and Methods” section. A comparison between the literature studies and ours is given in Table 9.

Our system achieved a 0.957 accuracy value for the first dataset, with limited data and computational load. The whole study, from image processing to classification, took only 3 h. With this study, we present a system that gives a high accuracy value with little time for those who have limited data and computational load.

## 6. Conclusions

This study presents a system to diagnose retinal diseases that has a high accuracy value with a low amount of data, time, and computing load. The main contribution of this study is not just the diagnosis of retinal diseases. The proposed system consists of basic methods that are used in artificial intelligence and image processing, so this system can be used for other problems to obtain better results with less data, time, and computing load. The proposed system achieves good results, but it achieves a slightly lower accuracy value for ‘AMD vs. DME’ classification in both datasets, which is acceptable as we consider the number of training data for both datasets. The purpose of the Firefly algorithm was to achieve results that were as good as the original data, with fewer features and less time, as mentioned before, so it fulfilled its duty, as proven in the results section. In this study, image processing is used to increase the accuracy value. There can be a decrease in the accuracy value for the ‘AMD vs. DME’ classification of the first dataset and the ‘Abnormal vs. Normal’ classification of the second dataset, but when we obtain the mean value of the classification results, we can say it increased the accuracy value. The code application was conducted in Google Colab notebooks. It has a 2.20 GHz Intel Xeon CPU, 12 GB of RAM, and 107.71 GB of disk space. Google Colab has GPU and TPU accelerators as well, but we did not use them, considering the purpose of this study.

## Figures and Tables

**Table 1:** Literature review of ML studies (Abbreviations: FE: Feature Extraction, FS: Feature Selection, CLS: Classifier, ACC: Accuracy).

Studies	Dataset			Number of Classes	Algorithms			Results
	Training	Testing	Total		FE	FS	CLS	
Liu et al. [21]	-	-	326	4 (one vs. rest)	LBP MSSP	PCA	SVM	0.93 AUC
Anantrasirichai et al. [22]	-	24	-	Unspecified	ILB CWT, etc.	PCA	SVM	0.8515 ACC
Alqudah et al. [23]	136,000	1250	137,250	5	AOCTNet	-	KNN	0.9944 ACC
Wang et al. [25]	-	-	2367	3	LCP + MSSP	-	SMO	0.9913 ACC
Albarrak et al. [26]	-	-	140	2	LBP HOG	PCA	Bayesian	0.914 ACC
Alsaih et al. [27]	-	-	4096	2	LBP HOG	PCA BoW	SVM	0.875 specificity

**Table 2:** Literature review of DL studies (Abbreviations: PU: Processing Unit, ACC: Accuracy).

Studies	Dataset		Number of Classes	Algorithms	Number of Layers	PU	Results (ACC)
	Training	Testing					
A P et al. [29]	83,484	968	4	OCTNet	29	GPU	0.9969
Fang et al. [30]	84,484	-	4	CNN	>15	GPU	0.8515
Das et al. [31]	84,484	-	4	DMF-CNN	>7	GPU	0.9603
Kim and Tran [35]	34,464	1000	4 (one vs. one)	UNet + FCN VGG16 VGG19 InceptionV3	-	CPU + GPU	0.983
Li et al. [36]	109,312	1000	4	VGG16	16	CPU + GPU	0.986
Asif et al. [37]	83,484	968	4	ResNet50	50	GPU	0.994
Tayal et al. [39]	75,269	1535	4	CNN	7	GPU	0.965

**Table 3:** The first dataset in the study.

Classification	ABNORMAL vs. NORMAL (CNV + DRUSEN + DME vs. NORMAL)		AMD vs. DME (CNV + DRUSEN vs. DME)		CNV vs. DRUSEN	
Class	ABNORMAL	NORMAL	AMD	DME	CNV	DRUSEN
Training	1200 + 1200 + 1200 = 3600	3600	2000 + 2000 = 4000	4000	3000	3000
Testing	81 + 81 + 80 = 242	242	121 + 121 = 242	242	242	242
Validation	200 + 200 + 200 = 600	600	400 + 400 = 800	800	600	600

**Table 4:** The second dataset in the study.

Classification	ABNORMAL vs. NORMAL (CNV + DRUSEN + DME vs. NORMAL)		AMD vs. DME (CNV + DRUSEN vs. DME)	
Class	ABNORMAL	NORMAL	AMD	DME
Training	473 + 851 = 1324	1157	473	473
Testing	75 + 75 = 150	150	150	150
Validation	50 + 50 = 100	100	100	100

**Table 5:** Firefly population example.

	F_1_	F_2_	F_3_	F_4_	…	F_33,000_
**f_1_**	0	1	0	0	1	1
**f_2_**	1	1	0	1	0	0
**f_3_**	0	1	0	1	1	1
**…**	1	1	1	1	1	0
**f_20_**	1	1	0	1	0	0

**Table 6:** Results of the study.

Dataset	Classifications	Metrics	Classes	SVM	LR	RF
First Dataset	Abnormal vs. Normal	Accuracy		0.967	**0.969**	0.923
		Precision	Abnormal	0.975	0.978	0.918
			Normal	0.959	0.959	0.928
		Recall	Abnormal	0.958	0.958	0.929
			Normal	0.975	0.979	0.917
		F1-Score	Abnormal	0.966	0.968	0.924
			Normal	0.967	0.969	0.923
	AMD vs. DME	Accuracy		0.909	**0.925**	0.851
		Precision	DME	0.93	0.947	0.912
			AMD	0.889	0.905	0.805
		Recall	DME	0.884	0.9	0.776
			AMD	0.933	0.95	0.925
		F1-Score	DME	0.906	0.923	0.839
			AMD	0.911	0.927	0.861
	CNV vs. DRUSEN	Accuracy		0.964	**0.977**	0.933
		Precision	CNV	0.959	0.971	0.91
			DRUSEN	0.97	0.983	0.96
		Recall	CNV	0.971	0.983	0.962
			DRUSEN	0.958	0.971	0.904
		F1-Score	CNV	0.965	0.977	0.935
			DRUSEN	0.964	0.977	0.931
	Mean Accuracy			0.946	***0.957***	0.902
Second Dataset	Abnormal vs. Normal	Accuracy		0.97	**0.976**	0.94
		Precision	Abnormal	0.966	0.955	0.902
			Normal	0.973	1	0.985
		Recall	Abnormal	0.973	1	0.986
			Normal	0.966	0.953	0.893
		F1-Score	Abnormal	0.9701	0.977	0.942
			Normal	0.969	0.976	0.937
	AMD vs. DME	Accuracy		**0.936**	0.933	0.91
		Precision	DME	0.887	0.882	0.855
			AMD	1	1	0.984
		Recall	DME	1	1	0.986
			AMD	0.873	0.866	0.833
		F1-Score	DME	0.94	0.937	0.916
			AMD	0.932	0.928	0.902
	Mean Accuracy			0.953	***0.954***	0.925

**Table 7:** Effect of the Firefly algorithm on accuracy and training time.

Dataset	Classifications	Metrics	Firefly Algorithm	SVM	LR	RF
First Dataset	Abnormal vs. Normal	Accuracy	Yes	0.967	0.969	0.923
			No	0.969	0.967	0.886
		Training time (s)	Yes	315.31	63.84	55.46
			No	452.92	81.4	68.38
	AMD vs. DME	Accuracy	Yes	0.909	0.926	0.851
			No	0.929	0.934	0.828
		Training time (s)	Yes	141.94	47.15	36.34
			No	307.82	86.83	52.67
	CNV vs. DRUSEN	Accuracy	Yes	0.965	0.977	0.934
			No	0.965	0.971	0.934
		Training time (s)	Yes	117.24	24.93	35.29
			No	308	64.17	61.06
Second Dataset	Abnormal vs. Normal	Accuracy	Yes	0.97	0.976	0.94
			No	0.973	0.976	0.953
		Training time (s)	Yes	11.71	7.17	9.33
			No	23.52	11.17	13.61
	AMD vs. DME	Accuracy	Yes	0.933	0.933	0.91
			No	0.936	0.927	0.916
		Training time (s)	Yes	9.36	5.2	4.23
			No	10.86	5.99	4.54

**Table 8:** Effects of image preprocessing on accuracy for both datasets.

Dataset	Classification	Image Preprocessing	SVM	LR	RF
First Dataset	Abnormal vs. Normal	Yes	0.967	0.969	0.923
		No	0.956	0.95	0.77
	AMD vs. DME	Yes	0.909	0.926	0.851
		No	0.923	0.931	0.762
	CNV vs. DRUSEN	Yes	0.964	0.977	0.933
		No	0.954	0.963	0.874
Second Dataset	Abnormal vs. Normal	Yes	0.97	0.976	0.94
		No	0.986	0.983	0.896
	AMD vs. DME	Yes	0.936	0.933	0.91
		No	0.88	0.863	0.72

**Table 9:** Comparison of the studies (Abbreviations: PU: Processing Unit, ACC: Accuracy, SPC: Specificity).

Studies	Dataset		Number of Classes	Algorithms			PU	Results
	Training	Testing		FE	FS	CLS		
A P et al. [29]	83,484	968	4	OCTNet	-	OCTNet	GPU	0.9969 ACC
Alqudah et al. [23]	136,000	1250	5	AOCTNet	-	KNN	CPU	0.9944 ACC
Asif et al. [37]	83,484	968	4	ResNet50	-	ResNet50	GPU	0.994 ACC
Wang et al. [25]	2367 total		3	LCP + MSSP	CFS	SMO	-	0.9913 ACC
Li et al. [37]	109,312	1000	4	VGG16	-	VGG16	CPU+ GPU	0.986 ACC
Kim and Tran [35]	34,464	1000	4 (one vs. one)	UNet + FCN VGG16 VGG19 InceptionV3	-	VGG16 VGG19 InceptionV3	CPU+ GPU	0.983 ACC
Tayal et al. [40]	75,269	1535	4	CNN	-	CNN	GPU	0.965 ACC
Das et al. [31]	84,484	-	4	DMF-CNN	-	DMF-CNN	GPU	0.9603 ACC
**Ours**	**13,600**	**968**	**4** **(hierarchy)**	**GLCM** **HOG** **VGG16** **DenseNet**	**Firefly Algorithm**	**LR**	**CPU**	**0.957 ACC**
Liu et al. [21]	326 total		4 (one vs. rest)	LBP MSSP	PCA	SVM	CPU	0.93 AUC
Albarrak et al. [26]	140 total		2	LBP HOG	PCA	Bayesian	CPU	0.914 ACC
Alsaih et al. [27]	4096 total		2	LBP HOG	PCA BoW	SVM	CPU	0.875 SPC
Fang et al. [30]	84,484	-	4	CNN	-	CNN	GPU	0.8515 ACC
Anantrasirichai et al. [22]	-	24	-	ILB CWT, etc.	PCA	SVM	CPU	0.8515 ACC

## Data Availability

The data used in this study are available at https://www.kaggle.com/datasets/naredlaajayreddy/oct-retina-images and https://people.duke.edu/~sf59/Srinivasan_BOE_2014_dataset.htm (accessed on 30 July 2022).

## Appendix A. Mathematical Expressions of Image Processing Techniques

The bilateral filter is different to the Gaussian filter. To see this difference with mathematical expressions, the Gaussian convolutional and bilateral filter operations are given in Equations (A1) and (A2) [41].

(A1) $$O_{p}=\sum_{q\in S}G_{\sigma }\left(\left||p-q|\right|\right)*I_{q}$$

(A2) $$O_{p}=\sum_{q\in S}G_{\sigma_{s}}\left(\left||p-q|\right|\right)*G_{\sigma_{r}}\left(\left|I_{P}-I_{q}\right|\right)*I_{q}$$

$O_{p}$ is the result of a filter in the p central pixel in both equations. S is a neighborhood that belongs to the p central pixel. The term ||p−q|| refers to the distance between the p central pixel and the q pixel. $I_{q}$ is the intensity value of q pixel. $G_{\sigma_{s}}$ is a spatial Gaussian kernel while $G_{\sigma_{r}}$ is a range Gaussian kernel. Gaussian kernel can be calculated as in Equation (A3) [41].

(A3) $$G_{\sigma }\left(x\right)=\frac{1}{2*\pi *\sigma^{2}}*e^{-\frac{x^{2}}{2*\sigma^{2}}}$$

The cumulative distribution function (cdf) is calculated according to Equation (A4). Histogram equalization formula from cdf, can be expressed as in Equation (A5) [48].

(A4) $$cdf\left(X_{k}\right)=\sum_{i=0}^{k}n_{i}$$

(A5) $$h\left(v\right)=round\left(\left(\frac{cdf\left(v\right)-cdf_{min}}{\left(M*N\right)-cdf_{min}}\right)*\left(L-1\right)\right)$$

## Appendix B. Mathematical Expressions of Feature Extraction Techniques

The mathematical expressions for GLCM features are given in Equations (A6)–(A9). μ refers to the mean of values, while σ refers to the standard deviation [49].

(A6) $$Energy=\sum_{i=0}^{L-1}\sum_{j=0}^{L-1}GLCM{\left[i,j\right]}^{2}$$

(A7) $$Correlation=\sum_{i,j=0}^{L-1}GLCM\left[i,j\right]\left[\frac{\left(i-\mu_{i}\right)*\left(j-\mu_{j}\right)}{\sqrt{\sigma_{i}^{2}*\sigma_{j}^{2}}}\right]$$

(A8) $$Homogeneity=\sum_{i=0}^{L-1}\sum_{j=0}^{L-1}\frac{GLCM\left[i,j\right]}{1+{\left(i-j\right)}^{2}}$$

(A9) $$Contrast=\sum_{i=0}^{L-1}\sum_{j=0}^{L-1}GLCM\left[i,j\right]{\left(i-j\right)}^{2}$$

The gradients’ ($G_{x}$, $G_{y}$) angle (θ) and magnitude (G) for Histogram of Gradients can be calculated as in Equations (A10) and (A11).

(A10) $$\theta \left(i,j\right)=\arctan\left(\frac{G_{y}\left(i,j\right)}{G_{x}\left(i,j\right)}\right)$$

(A11) $$G\left(i,j\right)=\sqrt{G_{x}{\left(i,j\right)}^{2}+G_{y}{\left(i,j\right)}^{2}}$$
